# Occurrence and Diversity of *Listeria monocytogenes* Isolated from Two Pig Manure Treatment Plants in France

**DOI:** 10.1264/jsme2.ME22019

**Published:** 2022-11-12

**Authors:** Martine Denis, Christine Ziebal, Evelyne Boscher, Sylvie Picard, Morgane Perrot, Meryl Vila Nova, Sophie Roussel, Arnaud Diara, Anne-Marie Pourcher

**Affiliations:** 1 ANSES, Unit of Hygiene and Quality of Poultry and Pork Products, Ploufragan, France; 2 INRAE, UR OPAALE, 17 Avenue de Cucillé-CS 64427, F-35044 Rennes, France; 3 ANSES, Unit Salmonella and Listeria, 14 Rue Pierre et Marie Curie, F-94700 Maisons-Alfort, France

**Keywords:** *Listeria monocytogenes*, pig, manure treatment, PFGE, MLST

## Abstract

The presence of *Listeria monocytogenes* in piggery effluents intended for irrigation crops may be a source of bacterial dissemination in agriculture. The occurrence and diversity of *L. monocytogenes* in the farm environment were examined in two pig manure treatment systems (S1 and S2). Samples collected over the course of one year consisted of manure, the liquid fraction of treated manure (lagoon effluent), and soil surrounding the lagoon. *L. monocytogenes* was enumerated using the Most Probable Number (MPN) method, serotyped by PCR, genotyped by pulsed-field gel electrophoresis (PFGE), and sequenced for multilocus sequence typing (MLST). *L. monocytogenes* was detected in 92% of manure samples and in approximately 50% of lagoon effluent and soil samples. Concentrations ranged between 5 and 10^3^ MPN 100‍ ‍mL^–1^. Serogroups IIa, IIb, and IVb were identified. Diversity was high with 44 PFGE profiles (252 isolates) and 17 clonal complexes (CCs) (96 isolates) with higher diversity in manure at site S1 supplied by four farms. Some PFGE profiles and CCs identified in manure or in pig feces from a previous study were also detected in lagoons and/or soil, reflecting pig *L. monocytogenes* circulation throughout the manure treatment and in the vicinity of the sampling sites. However, some PFGE profiles and CCs were only found in the lagoon and/or in soil, suggesting an origin other than pigs. The present study highlights the limited ability of biological treatments to eliminate *L. monocytogenes* from pig manure. The persistence of some PFGE profiles and CCs throughout the year in the lagoon and soil shows the ability of *L. monocytogenes* to survive in this type of environment.

Pig manure treatment technologies have been developed to limit the eutrophication of surface water originating from nonpoint agricultural sources. The most common treatment system in Brittany (France) consists of mechanically separating raw manure followed by a biological aerobic treatment of the liquid fraction, which is then stored in a tank or further separated into a thickened fraction and liquid effluent. The solid fraction resulting from the separation of raw manure is exported from the farm, while thickened treated manure is spread over the soil and the liquid effluent is stored in a lagoon before being used to irrigate crops. These treatment systems effectively remove nitrogen and phosphorus, but have a relatively weak impact on indicator bacteria (*Escherichia coli* and enterococci) and pathogenic bacteria (Pourcher *et al.*, 2012). Among the pathogenic bacteria that cause human foodborne illnesses, *Listeria monocytogenes* in pig feces (Beloeil *et al.*, 2003; Farzan *et al.*, 2010) is of particular concern given its capacity to survive in natural environments. *L. monocytogenes* has been detected in soil (Dowe *et al.*, 1997; Nightingale *et al.*, 2004; Locatelli *et al.*, 2013; Linke *et al.*, 2014) and water (Schaffter and Parriaux, 2002; Lyautey *et al.*, 2007; Cooley *et al.*, 2014; Linke *et al.*, 2014; Stea *et al.*, 2015). This pathogenic bacteria may survive for more than one month in soil (Jiang *et al.*, 2004; Nicholson *et al.*, 2005; Hutchison *et al.*, 2005; Vivant *et al.*, 2013; Genereux *et al.*, 2015; Moynihan *et al.*, 2015). Although *L. monocytogenes* has been detected in pig manure, the impact of treatments on the persistence of this bacterium is poorly documented. Two studies reported the fate of *L. monocytogenes* after its inoculation in manure. Nicholson *et al.* (2005) demonstrated that it survived for 4 days during the storage of pig farm manure. In a laboratory experiment by Grewal *et al.* (2007), a strain of *L. monocytogenes* inoculated in liquid pig manure (aerated or not) stored at 20–25°C decreased by 4–5 Log_10_ after 14 days and was no longer detected after 28 days. In a study of 44 piggeries in Brittany (France), Pourcher *et al.* (2012) showed the higher occurrence of *L. monocytogenes* in the effluent stored in lagoons after biological treatment (24% of samples) than in raw manure (18% of samples). This finding suggests that lagoons, which are used to irrigate crops, are favorable for the survival of *L. monocytogenes*. Isolates were not characterized in that study and the higher detection rate of *L. monocytogenes* in the lagoons was not explained. Due to their high discriminatory power, pulsed-field gel electrophoresis (PFGE) and multilocus sequence typing (MLST) has been used to subtype *L. monocytogenes* isolates from diverse compartments of the food chain (including food, the processing plant environment, and human infection), monitor the circulation of strains, and identify persistent strains that survive in the environment (Lopez-Valladares *et al.*, 2014; Li *et al.*, 2016; Ortiz *et al.*, 2015; Véghová *et al.*, 2017; Skowron *et al.*, 2018; Félix *et al.*, 2018).

To assess the role of manure treatments in the dissemination of *L. monocytogenes* in the environment, we herein examined the occurrence and concentration of *L. monocytogenes* and the PFGE profiles and clonal complexes (CCs) of isolates in raw manure, lagoon effluent, and the soil surrounding the lagoon in two pig manure treatment plants. The effects of the season and the physicochemical parameters of raw manure and lagoon effluents on the pre­valence of *L. monocytogenes* were also investigated.

## Materials and Methods

### Study sites

Samples were collected from two manure treatment plants (sites S1 and S2) in Brittany, France. The manure treatment system (Fig. S1) consisted of a storage tank discontinuously supplied with pig manure from four farms (site S1; only one storage tank for all four manures) and from one farm (site S2), followed by mechanical separation to concentrate phosphorus in the solid phase. The liquid phase was transferred to a nitrification-denitrification reactor. After a biological N treatment, the effluent was dehydrated with a filter band press (site S1) or transferred to a settling tank (site S2). The clarified effluent was discharged into a rectangular lagoon measuring 2,100 m^2^ at site S1 and 1,200 m^2^ at site S2. Raw manure was stored for 3–4‍ ‍weeks in the storage tank. The hydraulic retention time in the nitrification-denitrification reactor was approximately 40 days. The clarified effluent was stored in the lagoon for 10–12 months before being used to irrigate crops.

### Sampling

Between March 2012 and February 2013, samples were taken monthly from the manure storage tank and twice a month from both the lagoon and the soil surrounding the lagoon at each site. The three matrices (manure, lagoon effluent, and soil) were collected on the same day each month. Supplementary lagoon effluent and soil samples were collected at regular intervals between the monthly samples.

At each site visit, manure, lagoon effluent, and soil were sampled three times each in independent samples: (3×10 L) for liquid manure, (3×10 L) for the lagoon effluent, and (3×1–2 kg) for soil. Therefore, 36 samples of manure (3×12 sampling dates) and 57 samples of the lagoon effluent and of soil (3×19 sampling dates) were analyzed at each site. Manure samples were obtained after mixing the storage tank with a propeller. Each sample of the effluent lagoon was a mixture of four sub-samples, with each sub-sample being taken from each side of the lagoon (Fig. S1). Soil samples were collected in the first 5‍ ‍cm of topsoil located 5 to 10 meters from the lagoon. Each sample was immediately placed in a plastic bag (soil) or in a 1-L flask (manure or lagoon effluent). Samples for microbiological ana­lyses were stored at 4°C and analyzed within 24 h. Samples for chemical ana­lyses were stored at 4°C or frozen at –20°C.

### Physicochemical ana­lysis

Chemical parameters were measured for manure and lagoon effluent samples collected monthly. Chemical oxygen demand (COD) was measured using the standard NF T90-101 method (AFNOR, 1997). Anions and cations were measured by ionic chromatography. Volatile fatty acids (VFAs) were analyzed in soluble fractions by HPLC-UV. Total solids (TS), volatile suspended solids (VSS), total Kjeldahl nitrogen (TKN), total ammonia nitrogen (TAN), and pH were measured using standard methods (APHA, 1998). Copper and zinc ana­lyses were performed at the INRA Soil Analysis Laboratory in Arras (France). Temperature was measured monthly in manure and twice a month in the lagoons using a hand-held probe (ODEON X).

### Quantification of *L. monocytogenes*

*L. monocytogenes* was enumerated in each sample using the three-tube Most Probable Number (MPN) method. MPN dilution series were prepared by transferring 25, 2.5, 0.25, and 0.025‍ ‍mL of effluent (manure or lagoon) and 25, 2.5, and 0.25‍ ‍g of soil into ONE broth-*Listeria* medium (OBL). The two highest quantities were transferred into 225 and 22.5‍ ‍mL of OBL, respectively and the lowest quantities were initially diluted in OBL. One milliliter of the resulting dilution (containing 0.25‍ ‍mL of effluent, 0.25‍ ‍g of soil, or 0.025‍ ‍mL of effluent) was transferred into 9‍ ‍mL of OBL. After an incubation at 30°C for 48 h, a loopful of each broth was spread over Rapid L’mono agar (Bio-Rad) and incubated at 37°C for 48 h. Characteristic (blue) colonies of *L. monocytogenes* were identified based on their phosphatidylinositol phospholipase C (PIPLC) activity. Typical colonies of *L. monocytogenes* were isolated on Tryptose Soy-Yeast Extract (TSYE) plates (Biokar) and incubated at 37°C for 24 h. Purified isolates were stored at –80°C. An MPN tube was considered to be positive if a colony was confirmed by subsequent bacterial DNA extraction and PCR targeting *prfA* genes (as described below). MPN values were calculated using MPN Calculator Build 12 by Mike Curiale. The limit of detection was 1.4 MPN 100‍ ‍mL^–1^ or 100 g^–1^. Results are expressed in MPN 100‍ ‍mL^–1^ or 100 g^–1^.

In each visit, if the matrix was positive, a maximum of nine isolates in each matrix (three isolates×three samples in each matrix) were kept and stored in peptone glycerol broth at –80°C. A total of 252 isolates of *L. monocytogenes* were stored.

### Molecular serotyping (252 isolates)

Isolates of *L. monocytogenes* were serotyped using two successive PCRs, as described by Doumith *et al.* (2004) and Kerouanton *et al.* (2010). The first multiplex PCR was based on the amplification of the target genes, *prs*, *lmo*0737, *lmo*1118, ORF2110, ORF2819, and *prfA* (8). The *prs* and *prfa* genes confirmed that the isolate belonged to the genus *Listeria* and to *monocytogenes* species, respectively. Isolates presumed to belong to serogroup IIa were then submitted to a second PCR targeting the *flaA* gene (Kerouanton *et al.*, 2010) in order to confirm this serogroup. Serotyping assays clustered *L. monocytogenes* strains into five molecular serogroups: IIa (1/2a and 3a serovars), IIb (1/2b, 3b, and 7 serovars), IIc (1/2c and 3c serovars), IVa (4a and 4c serovars), and IVb (4ab, 4b, 4d, and 4e serovars). Four *L. monocytogenes* reference strains were used as positive controls for PCRs: ATCC 35152 (serogroup IIa), CIP 105449 (serogroup IIb), CIP 103573 (serogroup IIc), and ATCC13932 (serogroup IVb). One strain of *L. innocua* ATCC33090 was also used. All strains were purchased from the Pasteur Institute Collection (CIP), France. Isolates were sub-cultured on PCA plates (BioMerieux) at 37°C for 24 h. Some colonies from the bacterial culture were used for DNA extraction using the InstaGene® Matrix (BioRad) according to the manufacturer’s recommendations. DNA was adjusted to 10‍ ‍ng μL^–1^ and stored at –20°C until used.

The first multiplex PCR assay, adapted from that described by Doumith *et al.* (2004), was run using a Qiagen PCR multiplex kit (Qiagen) in a total volume of 25‍ ‍μL with 12.5‍ ‍μL of the mix and 1‍ ‍μL of the DNA extract. Twelve primers were used in the PCR reaction (lmo0737-1, lmo0737-2, lmo1118-1, lmo1118-2, orf2110-1, orf2110-2, orf2819-1, orf2819-2, prs-1, prs-2, lip-1, and lip-2 [Sigma Aldrich]) with a final concentration of 0.2‍ ‍μM in the PCR reaction for each primer. The amplification program began with a 15-min step at 95°C, followed by 35 cycles at 94°C for 30‍ ‍s, 60°C for 90‍ ‍s, and 72°C for 90‍ ‍s, and then a final step at 72°C for 90 s.

The second PCR, used to detect the presence of the *flaA* gene, was performed in a total volume of 25‍ ‍μL with 2‍ ‍μL of the DNA extract, 2.5‍ ‍μL of Faststart buffer without MgCl_2_ (1×) (Roche), 4‍ ‍μL of MgCl_2_ (Roche) with a final concentration of 4‍ ‍mM, 0.5‍ ‍μL of dNTPs (Ozyme) with a final concentration of 0.2‍ ‍mM, 1‍ ‍μL of each flaA primer (Sigma Aldrich) with a final concentration of 0.8‍ ‍μM each, and 0.2‍ ‍μL of FastStart Taq Polymerase (Roche) at 5‍ ‍U μL^–1^. The amplification program began with a 3-min step at 95°C, followed by 40 cycles at 94°C for 30‍ ‍s, 61°C for 40‍ ‍s, and 72°C for 1‍ ‍min 15‍ ‍s, with a final step at 72°C for 7‍ ‍min. Amplified PCR fragments were separated by 2% agarose gel electrophoresis in 1× TBE (Tris-Borate-EDTA) buffer (Gibco, Life Technologies) and visualized after GelRed staining (Biotum).

### Pulsed-field gel electrophoresis (PFGE) (252 isolates)

Bacterial cultures for DNA isolation were cultivated on tryptone soy agar with yeast extract (TSAYE) plates (OXOID) at 37°C for 24 h. Bacterial DNA was prepared by extracting chromosomal DNA according to the CDC PulseNet standardized procedure for typing *L. monocytogenes* (Graves and Swaminathan, 2001) and digested with two macrorestriction enzymes, *ApaI* and *AscI* (Biolabs). The first digestion was performed at 30°C for 6 h and the second at 37°C for 3 h. Restriction fragments were separated on a 1% SeaKem Gold agarose gel (Cambrex Bio Science) using the CHEF method in a CHEF-DRIII (Bio-Rad SA) apparatus. A linear ramping factor with pulse times ranging between 4.0 and 40.0‍ ‍s at 14°C was applied for 21 h with a voltage of 6‍ ‍V cm^–1^. *Xba*I-digested DNA from *Salmonella* Branderup H9812 (*Xba*I from Biolabs, at 37°C for 6 h) was included as a reference in all PFGE gels (Hunter *et al.*, 2005).

PFGE patterns were analyzed using BioNumerics software version 5.0 (Applied Maths). *ApaI* or *AscI* patterns were compared according to band positions with a maximum optimization at 1% using Dice band-based similarity coefficients with a maximum position tolerance of 1%. An *ApaI-AscI* PFGE profile was defined for each isolate by the combined results obtained with the two enzymes; they were coded Axx-Cyy (xx and yy corresponding to a number). The genetic diversity of *L. monocytogenes* populations was assessed by Simpson’s diversity index (D) with a 95% confidence interval as described by Hunter (1990) and Grundmann *et al.* (2001) using the online method available at http://www.comparingpartitions.info. The diversity indices of two populations were considered to be significantly different when the 95% confidence intervals linked to each index did not intersect.

The *ApaI-AscI* PFGE profiles obtained from manure and the lagoon effluent in the present study were also compared under BioNumeric with 75 profiles of 124 strains isolated from pig feces in a previous study (Boscher *et al.*, 2012) by the unweighted pair group method using averages (UPGMA). Strains were clustered together when *ApaI-AscI* PFGE profiles shared at least 90% similarity.

### DNA extraction and multi locus sequence typing (MLST) (96 isolates)

Ninety-six isolates representing the diversity of PFGE *Apa1*-*Asc1* profiles within each matrix (45 from site S1 and 51 from site S2) were selected for whole genome sequencing. DNA was extracted using the QIAamp DNA mini kit from Qiagen according to the manufacturer’s instructions and then used for library preparation with the Nextera XT library preparation kit. Sequencing was performed on the NextSeq 500 platform. NGS-based MLST predictions identified ST and CCs; when six out of seven MLST alleles were present, a CC was assigned when possible.

Reads were processed, assembled, and annotated through a harmonized in-house workflow called ARTwork (Assembly of Reads and Typing workflow) implemented in the ANSES Laboratory for Food Safety. This tool has been described in detail by Vila Nova *et al.* (2019) and is available at https://github.com/afelten-Anses/ARtWORK.

Normalized reads (*i.e.* 100×), scaffold assembly, and annotated genomes were stored in an in house genomic database in the ANSES Laboratory for Food Safety. Sequences with a mean coverage below 20× were excluded (*n*=2) as well as sequences with an abnormal assembly length (*i.e.* <200 bp) or poor assembly quality (*i.e.* >200 contigs). In addition, inter- and intra-species cross contaminations were evaluated using confindR software (V. 0.5.1) (Low *et al.*, 2019).

The first step in the prediction of pairwise Single Nucleotide Polymorphisms (SNPs), Insertions, and Deletions (InDels) from the mapping of reads against the reference Lm EGDe (HG813249.1) was produced by Snippy (V.4.6.0) (https://github.com/tseemann/snippy). The pseudogenome was then corrected for homologous recombination events with Gubbins (2.4.1) (Croucher *et al.*, 2014). The phylogenetic tree was ultimately calculated on the pseudogenome without recombination events based on the genomic distance using FastTree (V.2.9.1) (Price *et al.*, 2009). The final recombination tree was visualized by iTOL (Letunic and Bork, 2019).

### Statistical ana­lysis

Physicochemical and chemical parameters were subjected to an ana­lysis of variance (ANOVA). The distribution of data was tested with the Shapiro-Wilk test. Depending on the distribution of data (normally or not normally distributed), differences were tested using the Student-Newman-Keuls test or the non-parametric Kruskal-Wallis test and were considered to be significant at *P*<0.05. Pearson’s correlation coefficients were assessed for concentrations of *L. monocytogenes versus* the temperature and chemical parameters of manure and lagoon effluent samples. Statistical tests were performed using XLSTAT 2001 software.

## Results

### Physicochemical parameters in manure and lagoon effluent samples

Physicochemical ana­lyses were performed monthly on the two sites. The mean values of the physicochemical parameters in raw manure and lagoon effluent samples at sites S1 and S2 are listed in Table 1. The characteristics of raw manure were similar. TS, VSS, COD, and TKN were less concentrated in manure S1 than in manure S2, whereas ion and metal contents were of the same order of magnitude in both. Except for Na^+^, Ca^++^, and K^+^, the composition of the lagoon effluent significantly differed from that of manure at both sites. Over the study period, TS, COD, and TKN in the lagoon effluent remained relatively stable, whereas the pH of the two lagoons varied with the season; the maximum value (pH 9.2) was observed in summer and the minimum in winter (pH 8.3) (Fig. S2). Regardless of the matrix, temperatures at S1 and S2 fluctuated in a similar manner, ranging between 6.0 and 20.2°C in manure storage tanks and between 3.6 and 20.7°C in the lagoons depending on the season (Fig. S3). The centrifugation of raw manure followed by the biological treatment and phase separation (pressing at site S1 and settling at site S2) led to an overall reduction in TS (80% at site S1 and 78.3% at site S2), VSS (97.7 and 96.3%), COD (96.9 and 91.5%), TKN (97.7 and 95.4%), NH_4_^+^ (>99 and 94.7%), Cu (97.8 and 92.4%), and Zn (99.6 and 92.7%).

### Concentrations of *L. monocytogenes* in manure, lagoon effluent, and soil samples

**Fig. 1.**
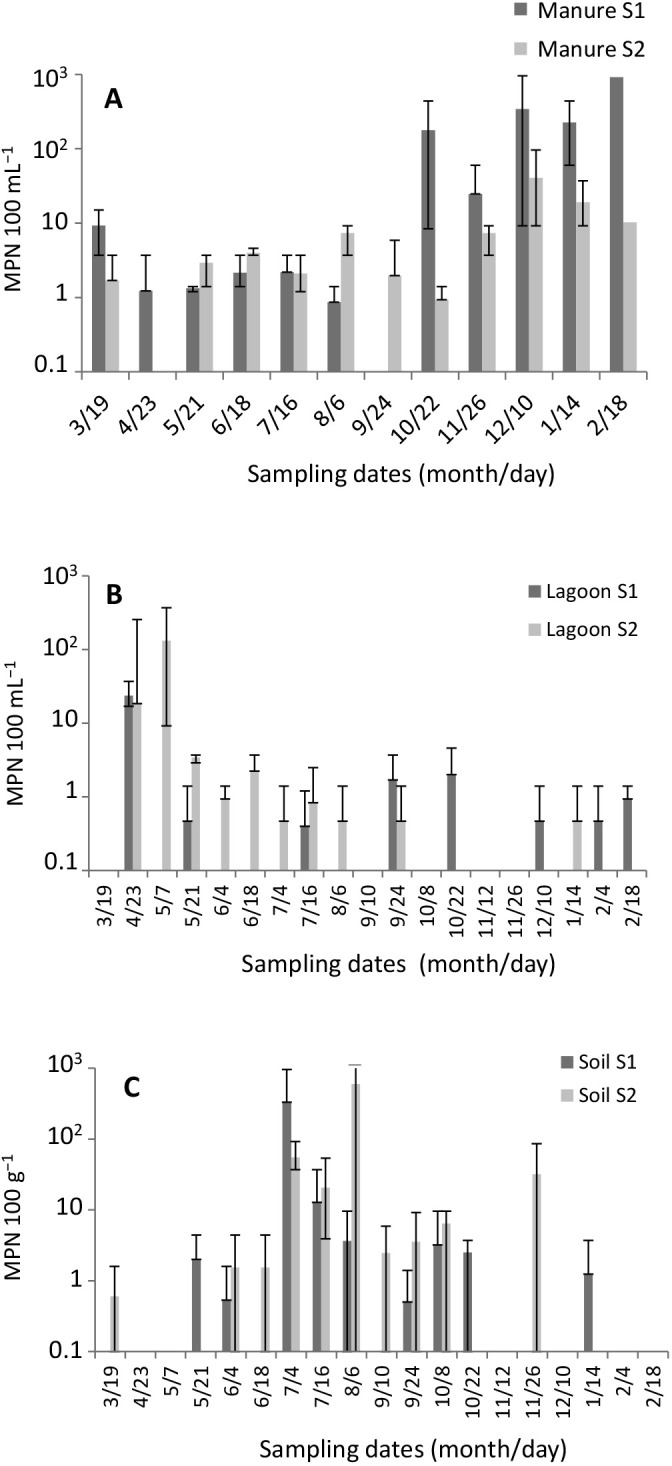
Average concentration of *Listeria monocytogenes* (*n*=3 per sampling date) in manure (A), lagoon effluent (B), and soil (C) in two manure treatment systems (S1 and S2) over a period of one year. Bars indicate minimum and maximum values.

*L. monocytogenes* was detected in 92% of manure samples, regardless of the site, in 42% of lagoon effluent samples at site S1 and 53% at site S2, and in 47% of soil samples at site S1 and 53% at site S2. The distribution of positive samples according to the matrix did not significantly differ between the two sites (chi-squared, *P*=0.56). When detected, *L. monocytogenes* concentrations ranged between 5 and 10^3^ MPN 100‍ ‍mL^–1^ regardless of the matrix. *L. monocytogenes* was present throughout the year, with the highest levels being observed in manure in winter, in lagoon effluents in spring, and in soil samples in summer (Fig. 1). The concentrations of *L. monocytogenes* reached 9.2×10^2^ and 4.1×10^1^ MPN 100‍ ‍mL^–1^ in manure at sites S1 and S2, respectively. In the lagoons, concentrations were <4 MPN 100‍ ‍mL^–1^, except for three samples: two that were collected from lagoons S1 and S2 in April (2.3×10^1^ MPN 100‍ ‍mL^–1^ and 1.8×10^1^ MPN 100‍ ‍mL^–1^, respectively), and one collected from lagoon S2 in May (1.3×10^2^ MPN 100‍ ‍mL^–1^). The average reduction in the level of *L. monocytogenes* between manure and lagoon samples was 1.5 Log_10_ at site S1 and 0.3 Log_10_ at site S2. The concentrations of *L. monocytogenes* correlated with two of the 15 chemical and physicochemical parameters: the temperature of manure and the VSS level in the lagoon effluent (Pearson’s correlation coefficients of –0.50 and 0.46 respectively). No relationship was observed between *L. monocytogenes* and the other physicochemical parameters analyzed in the present study (Table S1 and S2). *L. monocytogenes* levels in soil reached 3.3×10^2^ MPN 100 g^–1^ in soil at site S1 in July and 6.0×10^2^ MPN 100 g^–1^ in soil at site S2 in August.

### *L. monocytogenes* serogroups and PFGE profiles

A total of 252 *L. monocytogenes* isolates were characterized by PCR to identify their serogroups and typed by PFGE with *ApaI* and *AscI* restriction endonucleases. We considered the distribution of the serotypes and PFGE profiles within the matrices at both sites. However, it is important to note that this distribution may be slightly biased because between one and nine isolates in each matrix were examined in each visit.

The isolates were distributed among three serogroups: serogroup IIa (25% of isolates), IIb (28.6%), and IVb (46.4%), and their proportion differed between the two sites. Each serogroup was dominant in a particular matrix: serogroup IIa in manure (46.7 and 31.8% of isolates at sites S1 and S2, respectively), serogroup IIb in lagoon effluent (62.1 and 57.8%), and serogroup IVb in soil (43.8 and 90.5%).

The 252 *L. monocytogenes* isolates were distributed among 44 *ApaI-AscI* PFGE profiles. Of these, 4.3% of isolates (11) were represented by a single *ApaI-AscI* PFGE profile. Eighteen PFGE profiles occurred at least five times during the one-year survey, and accounted for 79.4% of all isolates. The diversity of profiles was higher in serogroup IIb (D=0.916‍ ‍_CI95%_ [0.88–0.95]), which was close to the diversity of profiles in serogroup IIa (D=0.883‍ ‍_CI95%_ [0.84–0.93]), but differed from that observed in serogroup IVb (D=0.808‍ ‍_CI95%_ [0.75–0.87]).

**Fig. 2.**
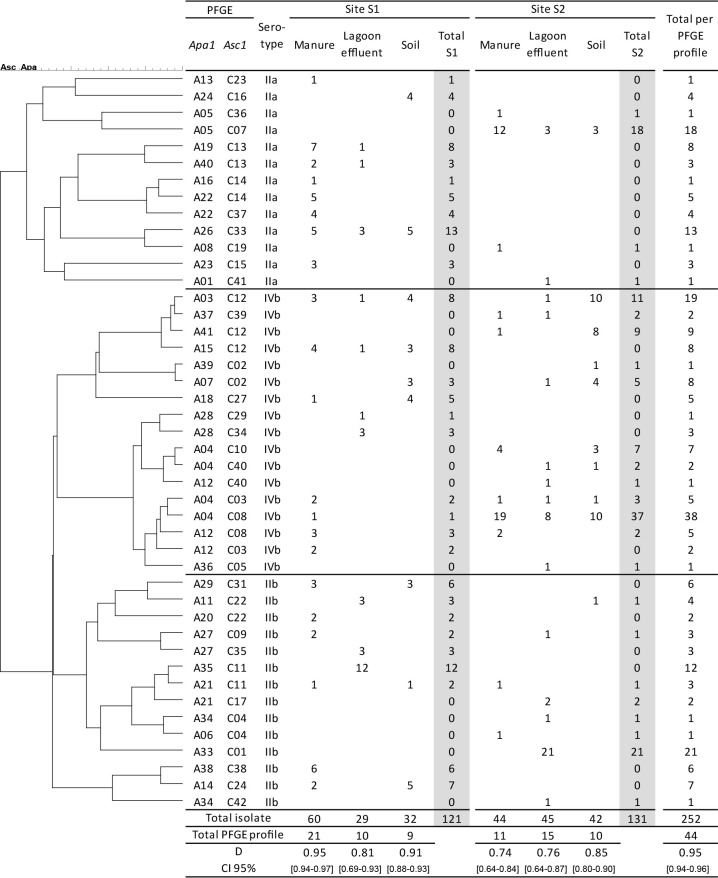
Distribution of isolates (*n*=252) at two sites and in three matrices according to their *Apa1-Asc1* PFGE profiles and index of diversity.

The dendrogram in Fig. 2 shows the distribution of *ApaI-AscI* PFGE profiles and the index of diversity of *L. monocytogenes* populations in each matrix. Twenty-eight *ApaI-AscI* PFGE profiles were detected at site S1, and 24 at site S2. Differences in diversity were observed between the two manures, with the highest diversity being detected in manure at S1 and the lowest in manure at S2. In manure at S2, two PFGE profiles represented 70% of isolates, whereas in manure at S1, the two most predominant profiles represented 21% of isolates. No significant differences were observed in diversity between isolates from lagoon effluents at S1 and S2 or between isolates from soil at S1 and S2 (Fig. 2).

Eight PFGE profiles were common to both sites. Twenty-four PFGE profiles were found in only one matrix (11 in manure, 11 in lagoon effluent, and two in soil). Six profiles (three at each site) were common to manure, lagoon effluent, and soil. Among the 17 PFGE profiles found in soil, nine were also present in another matrix (three in lagoon effluents and six in manure). Twelve PFGE profiles were detected at least twice in the same matrix (data not shown). Among them, one profile (A04C08) was isolated at multiple sampling points at site S2 and once in manure at site S1.

### Comparison of PFGE profiles with those identified in pig feces

The 44 *ApaI-AscI* PFGE profiles obtained from manure and lagoon effluents in the present study were compared under BioNumeric with 75 *ApaI-AscI* PFGE profiles of 124 strains isolated from pig feces in a previous study (Boscher *et al.*, 2012). Taking manure and lagoon effluent together, 57.7% (15/26, site S1) and 45.5% (10/22, site S2) of the PFGE profiles shared at least 90% similarity with the PFGE profiles of strains isolated from pig feces. Among the 23 profiles common to feces and manure or lagoon effluents, seven were also found in soil.

### Clonal complexes (CCs) and comparisons with those identified in pig feces

**Fig. 3.**
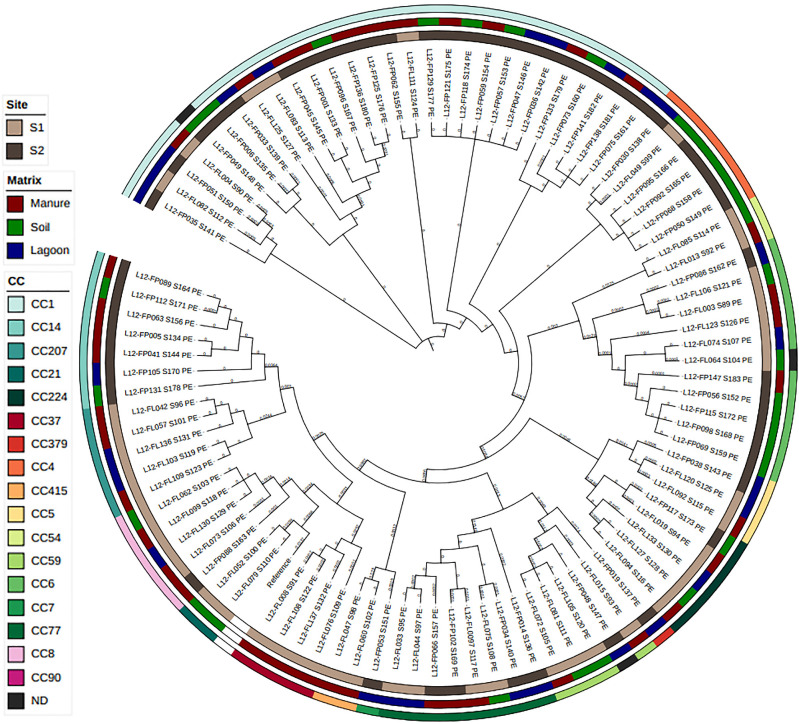
Phylogenetic distribution of isolates (*n*=96) at two sites and in three matrices according to their CCs.

Among 96 isolates, the allele profile was unknown (a presumable novel ST) or incomplete for three isolates (1 from site S1 and 2 from site S2). No CCs were identified for these three isolates (Table 2). The other 93 isolates were distributed among 17 CCs (14 at site S1 and 10 at site S2), with 7 common CCs between the two sites (CC1, CC4, CC5, CC6, CC8, CC77, and CC224). Their distributions are shown in a phylogenetic tree (Fig. 3). Global diversity was higher in S1 (D=0.94‍ ‍_CI95%_ [0.92–0.96]) than in S2 (D=0.78‍ ‍_CI95%_ [0.68–0.87]). Higher diversities were also observed for the three matrices, manure, lagoon effluent, and soil, at S1 than for the same matrices at S2 (Table 2).

Six CCs (CC7, CC21, CC37, CC90, CC379, and CC415) were observed in only one matrix (three in manure, two in lagoon effluent, and one in soil). Six CCs (CC1, CC6, CC8, CC14, CC77, and CC224) were common to manure, lagoon effluent, and soil. Among the 10 CCs found in soil, nine were also present in another matrix (seven in lagoon effluent and eight in manure).

The 17 CCs identified in the present study were compared to CCs identified by Félix *et al.* (2018) from our *L. monocytogenes* collection isolated in pig feces at a farm in a previous study (Boscher *et al.*, 2012). Thirteen of these 17 CCs were previously found in pig feces (CC1, CC4, CC5, CC6, CC7, CC8, CC14, CC21, CC37, CC59, CC77, CC90, and CC224) and 11 were common with CCs in lagoons and/or in soil in this study.

## Discussion

### Physicochemical characteristics of manure and lagoon effluent samples

The physicochemical characteristics of pig manure collected in the two storage tanks were similar to those reported in previous studies (Béline *et al.*, 2004; Carrère *et al.*, 2009; McLaughlin *et al.*, 2009; Viancelli *et al.*, 2013; McCarthy *et al.*, 2015). The reductions observed in chemical components throughout manure treatment systems is consistent with that reported elsewhere for this type of manure treatment (Béline *et al.*, 2004; Viancelli *et al.*, 2013). As reported by Béline *et al.* (2004), the mechanical separation of treated manure (filter press) was more efficient than settling, leading to a clarified effluent with only 2% of total nitrogen from raw manure, 3% of COD, and 0.4 to 2% of heavy metals.

### Occurrence and concentration of *L. monocytogenes*

The occurrence of *L. monocytogenes* in manure (Le Maréchal *et al.*, 2019) and soil and water (Sauders *et al.*, 2012; Linke *et al.*, 2014) was previously reported; however, limited information is currently available on the concentration of this pathogenic bacterium. The concentration of *L. monocytogenes* in the manure of two pig farms was previously shown to range between <10 CFU g^–1^ and 3.3×10^2^‍ ‍CFU‍ ‍g^–1^ (Le Maréchal *et al.*, 2019), which is of the same order as that in the present study. It is noteworthy that at the laboratory scale, *L. monocytogenes* has a relatively short survival time. Grewal *et al.* (2007) and Desneux *et al.* (2016), who examined the fate of inoculated strains in pig manure stored at 20–25°C, observed a Log_10_ reduction of >4.7 after 42 days of storage. The difference in the persistence of *L. monocytogenes* in flasks and at the farm scale may be explained by the bias associated with the inoculation of strains, the persistence of which may differ from that of autochthonous bacteria. Moreover, laboratory experiments were performed in batch, whereas at the farm scale, tanks are regularly fed with fresh manure contaminated with *L. monocytogenes*.

In the present study, cultivable *L. monocytogenes* was detected almost systematically in raw manure samples and in approximately 50% of lagoon effluent and soil samples throughout the one-year period. Since a viable, but non-cultivable state was previously highlighted in pig manure and lagoon effluent (Desneux *et al.*, 2016), the level of viable *L. monocytogenes* may have been underestimated in this study. Nevertheless, its prevalence was higher than that observed in other farm and non-farm environments. Fox *et al.* (2009) reported a prevalence of 19% in 298 fecal, silage, water, soil, and milk samples collected from 16 cattle farms. Linke *et al.* (2014) showed a prevalence of *L. monocytogenes* of 6 and 0% in soil and water environmental samples, respectively, in Austria. In another study, Nightingale *et al.* (2004) noted a prevalence of 24% on 16 cattle farms and 6% on 12 goat and sheep farms. In the present study, the level of *L. monocytogenes* reached 10^3^‍ ‍MPN 100‍ ‍mL^–1^ in raw manure and 10^2^ MPN 100‍ ‍mL^–1^ in lagoon effluent. The concentration of *L. monocytogenes* in the lagoons was close to the method’s limit of detection, which may also explain its irregular detection in lagoon effluent. The highest concentration of *L. monocytogenes* was measured in manure during the coldest months. This is consistent with the data reported by Boscher *et al.* (2012) showing a higher frequency of detection of *L. monocytogenes* in pig feces in the fall/winter season.

The inactivation of *L. monocytogenes* during the biological treatment of manure is due to the combined effects of biotic and abiotic factors. Erickson *et al.* (2014) reported that besides temperature, pH and ammonia levels influence reductions in the concentration of *L. monocytogenes* inoculated into compost mixtures. In the present study, the high pH observed in lagoons was not inhibitory because *L. monocytogenes* survives well under alkaline conditions (Sharma *et al.*, 2003) and is not affected by pH 9.0 (Cheroutre-Vialette *et al.*, 1998). Furthermore, neither VFA nor ammonia levels correlated with the concentrations of *L. monocytogenes* in the present study, suggesting that other physicochemical factors (*e.g.* VSS levels) or meteorological conditions (*e.g.* UV) inhibited *L. monocytogenes* in lagoons. It is important to note that the average levels of NH_4_^+^ (≤110‍ ‍mg L^–1^) and VFA (≤6‍ ‍mg L^–1^) in lagoons appeared to be too low to influence the survival of *L. monocytogenes*.

Although low temperatures favor the survival of *L. monocytogenes* (Jiang *et al.*, 2004; Sidorenko *et al.*, 2006), the highest level of pathogenic bacteria in the soil surrounding lagoons was measured during the warmest months. Since the level and occurrence of *L. monocytogenes* in lagoons were not particularly high in the summer, this result suggests that soil may have been contaminated by wildlife in this season. *L. monocytogenes* has been isolated from wild animals, including birds (Yoshida *et al.*, 2000; Kalorey *et al.*, 2006; Lyautey *et al.*, 2007; Hellstrom *et al.*, 2010; Sauvala *et al.*, 2021). It is noteworthy that the manure treatment did not result in a marked reduction in the level of *L. monocytogenes*, particularly at site S2 at which the concentration in the lagoon effluent was of the same order of magnitude as that of manure. These results suggest that *L. monocytogenes* was able to persist throughout the manure treatment process or that the lagoon had been contaminated by wildlife. The presence of *L. monocytogenes* in the soil surrounding the lagoons at concentrations similar to or even higher than those in the lagoons themselves suggests that pathogenic bacteria circulate in the environment of the treatment system. The circulation of *L. monocytogenes* in the farm environment has already been reported by Ho *et al.* (2007), who observed similar *L. monocytogenes* ribotypes in the feces of dairy cows as well as in silage.

### Characterization of strains

*L. monocytogenes* strains were assigned to serogroups IIa, IIb, and IVb based on the serotyping scheme described by Kerouanton *et al.* (2010). These serogroups mainly include serotype 1/2a, serotype 1/2b, and serotype 4b, respectively (Doumith *et al.*, 2004), which are frequently involved in outbreaks of listeriosis in humans (Goulet *et al.*, 2008). The presence of these three serogroups at the two sites is consistent with the predominance of serotypes 1/2a, 1/2b, and 4b (Boscher *et al.*, 2012) in pig feces and piggery effluent (Pourcher *et al.*, 2012). Serogroup IIa (serotypes 1/2a and 3a) is commonly found in food and appears to be widely distributed in natural and farm environments (Lomonaco *et al.*, 2015). We found a predominance of serogroup IIa in manure, of serogroup IIb in lagoon effluent, and of serogroup IVb in soil. Since the method of detection of *L. monocytogenes* was similar for the three matrices, the occurrence of the serogroups cannot be explained by a bias in the culture method, as suggested by Cooley *et al.* (2014). Therefore, the distribution observed in the different matrices indicates that these serogroups have different behaviors in these matrices. Soni *et al.* (2014) also reported the predominance of serotype 4b, 4d, or 4e in soil samples, while Linke *et al.* (2014) found that the majority of isolates in soil were serotypes 1/2a and 3a, and the remainder were serotypes 4b, 4d, 4e, 1/2b, and 3b.

PFGE and MLST were used to examine the dissemination of *L. monocytogenes* in the environment of manure treatment systems. As previously observed in sewage sludge and pig feces (Kerouanton *et al.*, 2010; Boscher *et al.*, 2012; Félix *et al.*, 2018), PFGE and MLST revealed high diversity among the *L. monocytogenes* isolates tested. Castro *et al.* (2018) also reported marked diversity among *L. monocytogenes* isolated from three dairy farms. Diversity was significantly higher in manure at site S1 (21 PFGE profiles, 12 CCs) than at site S2 (11 PFGE profiles, 5 CCs). This difference may be explained by the number of effluents that supply the storage tanks at the two sites: site S1 received four effluents, whereas site S2 received only one. The great diversity of PFGE profiles and CCs observed in the soil surrounding the lagoons confirmed the prevalence of *L. monocytogenes* in these environments (Soni *et al.*, 2014).

Eight PFGE profiles and 7 CCs were detected at both sites, which were located 10‍ ‍km from each other, suggesting that these strains were common in the geographic area examined. However, strains occurring in a predominant pattern or CCs at one site were not systematically found at the other site. Four PFGE profiles and 4 CCs were common to manure at both sites, suggesting that pigs from different farms excrete strains with the same PFGE types or CCs of *L. monocytogenes* as previously observed in pig farms (Boscher *et al.*, 2012; Félix *et al.*, 2018). It is important to note that among the PFGE profiles and CCs detected in lagoon effluent samples only, four strains and three strains had their PFGE profiles or CC, close (>90% similarity) or in common, respectively, with those of strains isolated from pig feces (Boscher *et al.*, 2012; Félix *et al.*, 2018). These results suggest that lagoon effluent is a favorable environment for the survival of some strains of *L. monocytogenes* originating from pigs. Furthermore, the dominant PFGE profile found in each lagoon effluent was not detected in manure or soil, reflecting the ability of specific strains of *L. monocytogenes* to adapt to this environment. This adaptation in lagoons may explain the small reduction observed in concentrations between manure and lagoon effluents. Four ST (ST 4, ST6, ST21, and ST59) previously reported in soil (Linke *et al.*, 2014) were also detected in soil in the present study, suggesting the ability of some CCs to survive in this environmental matrix. Nine PFGE profiles and 6 CCs were common to manure and lagoon samples, five PFGE and 5 CCs of which were also found in soil. Moreover, regardless of the matrix, some PFGE profiles and CCs were detected several times. Distinct PFGE profiles and CCs isolated from manure persisted over the one-year period, suggesting that some strains belonging to certain PFGE profiles or CCs continuously circulated in the vicinity of manure treatment systems. Furthermore, PFGE profiles and CCs were found in both pig manure and soil, which confirmed that they were widely distributed (Lyautey *et al.*, 2007). PFGE profiles and CCs detected only in soil may reflect the wild origin of some strains. Previous studies reported that wild birds and mammals harbor *L. monocytogenes* (Lyautey *et al.*, 2007; Hellstrom *et al.*, 2010; Wacheck *et al.*, 2010). ST1 and ST224 detected in the lagoon and soil, respectively, in the present study were recently identified in wild birds (Sauvala *et al.*, 2021).

This one-year survey revealed the constant presence in pig manure of cultivable *L. monocytogenes* belonging to serogroups involved in outbreaks of listeriosis in humans. The present results also highlight the limited ability of biological treatments to eliminate *L. monocytogenes*. While the presence of *L. monocytogenes* in manure may be due to its carriage by pigs, its presence in lagoon effluent may reflect its ability to survive biological treatments. Its presence in soil surrounding the lagoons and in lagoon effluent used for irrigation is a potential source of dissemination in the environment. However, limited information is currently available on interactions between wildlife and the environment of treatment processes because ana­lyses of PFGE profiles and CCs revealed that some PFGE types and CCs found in the effluent of two different lagoons did not appear to originate in pig manure.

## Citation

Denis, M., Ziebal, C., Boscher, E., Picard, S., Perrot, M., Nova, M. V., et al. (2022) Occurrence and Diversity of *Listeria monocytogenes* Isolated from Two Pig Manure Treatment Plants in France. *Microbes Environ ***37**: ME22019.

https://doi.org/10.1264/jsme2.ME22019

## Supplementary Material

Supplementary Material

## Figures and Tables

**Table 1.** Average values for chemical and physicochemical parameters in manure and lagoon effluent at sites S1 and S2 over a one-year period.

	Manure at S1 Mean±SD	Manure at S2 Mean±SD	Lagoon at S1 Mean±SD	Lagoon at S2 Mean±SD
pH	7.7±0.1^a^*	7.8±0.3^a^	8.9±0.3^b^	8.9±0.2^b^
TS g L^–1^	28.8±6.2^b^	41.0±7.6^a^	5.6±0.4^c^	8.9±1.1^c^
VSS g L^–1^	13.1±4.2^b^	24.6±5.3^a^	0.3±0.3^c^	0.9±0.2^c^
TKN gN L^–1^	3.0±0.4^bc^	4.15±0.4^c^	0.07±0.04^a^	0.19±0.07^ab^
COD gO_2_ L^–1^	29.0±6.5^b^	36.5±9.1^a^	0.9±0.1^c^	3.1±0.5^c^
VFA g L^–1^	0.93±0.85^b^	0.17±0.20^ab^	0.02±0.05^a^	nd^†^
PO_4_^–^ gP L^–1^	0.04±0.02^a^	0.06±0.04^ab^	0.09±0.04^b^	0.08±0.06^b^
Mg^++^ g L^–1^	0.04±0.02^a^	0.01±0.01^b^	0.07±0.04^b^	0.06±0.04^b^
Ca^++^ g L^–1^	0.19±0.06^b^	0.07±0.10^a^	0.04±0.03^a^	0.05±0.05^a^
K^+^ g L^–1^	2.0±0.2^a^	2.3±0.37^a^	2.5±1.1^a^	2.6±1.5^a^
Na^+^ g L^–1^	0.65±0.07^a^	0.63±0.05^a^	0.83±0.39^a^	0.61±0.48^a^
NH_4_^+^ g L^–1^	1.45±0.77^b^	1.9±0.9^b^	0.006±0.01^a^	0.10±0.27 ^a^
Cu mg L^–1^	16.7±4.5^a^	15.8±3.0^a^	0.36±0.05^b^	1.2±0.14^b^
Zn mg L^–1^	56.1±14.8^a^	49.4±6.3^b^	0.22±0.08^c^	3.6±0.42^c^

* Within a line, those with a different letter significantly differ (*P*<0.05) (the Newman-Keuls test or Kruskal Wallis test); ^†^ not detected

**Table 2.** Distribution of isolates at two sites and in three matrices according CCs and the index of diversity

matrix	Site S1					Site S2			
	manure	lagoon	soil	Total		manure	lagoon	soil	Total
CC1	3	2		5		9	7	6	22
CC4			1	1			1	4	5
CC5	1	1		2			1		1
CC6	2	1	1	4		1	1	4	6
CC7							1		1
CC8	2	1	1	4		1			1
CC14						4	1	2	7
CC21			2	2					
CC37	4			4					
CC54	1		1	2					
CC59	2		2	4					
CC77	1	2	1	4		2	2		4
CC90	1			1					
CC207	3	2		5					
CC224	2	1	1	4				1	1
CC379							1		1
CC415	2			2					
nd			1	1			1	1	2
Total	24	10	11	45		17	16	18	51
ID	0.94	0.93	0.96	0.94		0.68	0.82	0.82	0.78
_CI95%_	[0.91–0.97]	[0.87–1.00]	[0.91–1.02]	[0.92–0.96]		[0.50–0.87]	[0.64–1.00]	[0.73–0.90]	[0.68–0.87]

ID: Diversity index; CI: confidence interval at 95%.
