# Supplementary material for: Occurrence and Diversity of Listeria monocytogenes Isolated from Two Pig Manure Treatment Plants in France
Source: Microbes Environ. 2022 Nov 12;37(4):ME22019. doi: 10.1264/jsme2.ME22019 (PMC9763045; doi:10.1264/jsme2.ME22019)
Supplement: Supplementary file 1 — Supplementary Material [file 37_22019_s1.pdf]

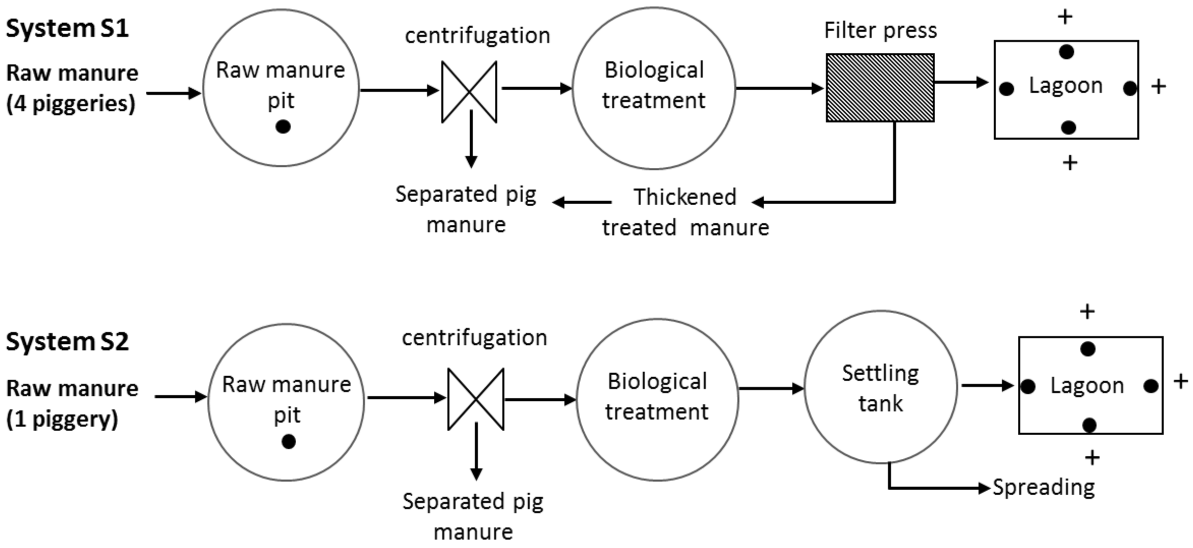

Figure S1. Operational flow of the two treatment systems.
Black dots and crosses indicate the sampling points of manure, lagoon effluent and soil, respectively.

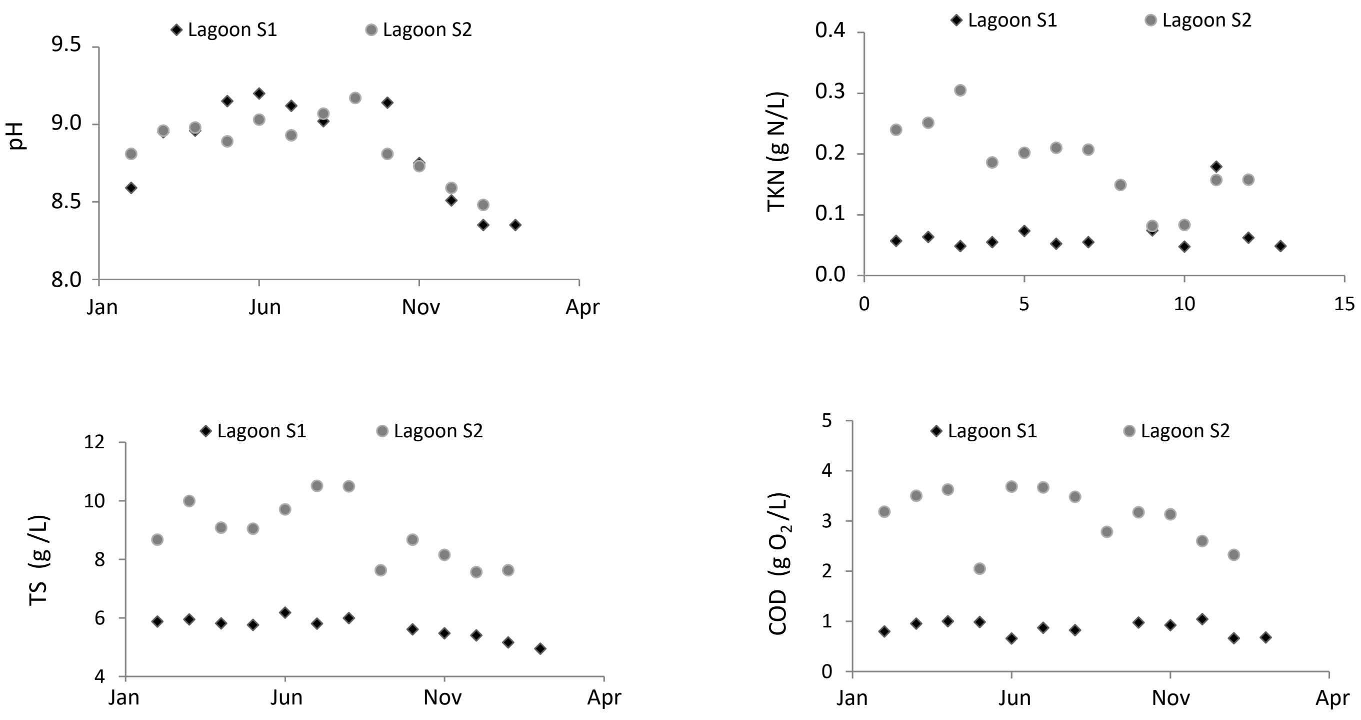

Figure S2. Evolution of the pH, TS, TKN and COD in lagoon effluents during the sampling period.

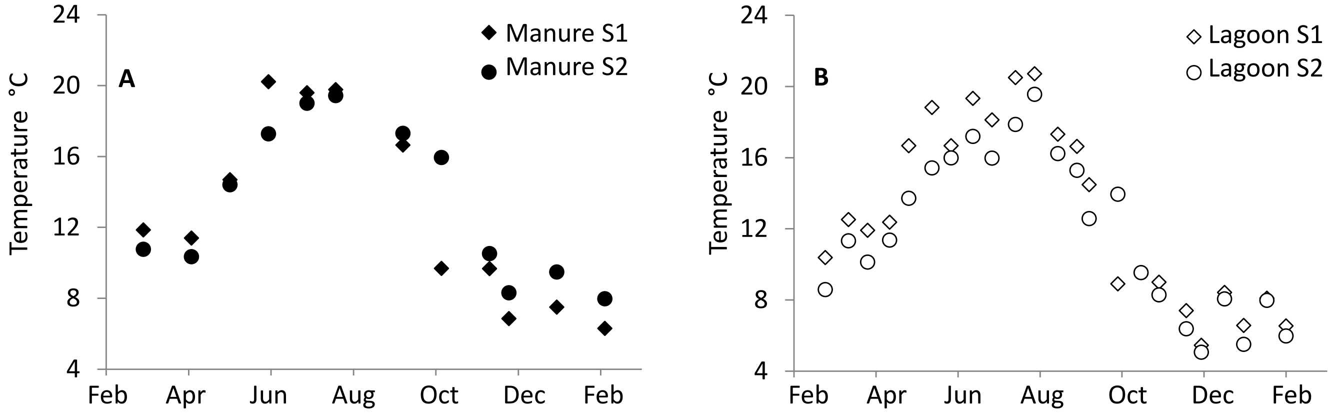

Figure S3 . Temperature of the manure in the storage tanks (A) and of the lagoon effluents (B) of the two manure treatment systems over the sampling period

TABLE S1. Pearson correlation matrix for the physicochemical properties of manures and the concentration of *L. monocytogenes*

	pH	TS	VSS	COD	TKN	NH_4^+	VFA	PO_4^{3-}	Mg^{2+}	Ca^{2+}	K^+	Na^+	Cu	Zn	temp	L. mono
pH	**1**															
TS	-0.07	**1**														
VSS	0.13	**0.89**[c]	**1**													
COD	-0.26	**0.66**	**0.60**	**1**												
TKN	-0.09	**0.73**	**0.76**	**0.59**	**1**											
NH_4^+	0.25	0.04	0.27	0.21	0.16	**1**										
VFA	0.22	-0.21	-0.29	-0.20	**-0.47**	-0.22	**1**									
PO_4^{3-}	-0.17	**0.46**	0.40	0.38	**0.47**	-0.25	**-0.42**	**1**								
Mg^{2+}	-0.13	-0.27	-0.40	-0.11	**-0.50**	-0.17	0.27	-0.39	**1**							
Ca^{2+}	-0.31	-0.10	-0.24	0.01	-0.34	-0.23	0.33	-0.20	**0.87**	**1**						
K^+	-0.07	-0.01	0.08	-0.02	0.34	0.11	-0.35	0.04	**-0.60**	**-0.66**	**1**					
Na^+	-0.04	-0.14	-0.20	-0.11	-0.06	-0.28	**0.58**	**-0.43**	0.13	0.17	0.27	**1**				
Cu	-0.16	0.35	0.16	0.25	0.09	-0.11	0.10	0.04	0.09	-0.01	-0.32	-0.05	**1**			
Zn	-0.21	0.25	0.08	0.34	0.04	-0.01	0.20	-0.14	0.32	0.23	-0.37	0.11	**0.88**	**1**		
temp[a]	0.09	0.08	0.16	**0.46**	0.28	**0.54**	-0.24	-0.11	0.22	0.04	-0.03	-0.16	0.20	0.39	**1**	
L. mono[b]	-0.16	-0.22	-0.25	-0.30	-0.39	-0.34	0.13	-0.18	0.18	0.22	-0.06	0.07	-0.08	-0.05	**-0.50**	**1**

[a] Temperature; [b] *L. monocytogenes*; [c] Numbers in bold indicate a significant correlation ($\alpha = 0.05$)

TABLE S2. Pearson correlation matrix for the physicochemical properties of lagoons and the concentration of *L. monocytogenes*

	pH	TS	VSS	COD	TKN	NH_4^+	VFA	PO_4^{3-}	Mg^{2+}	Ca2$^+$	K^+	Na^+	Cu	Zn	temp	L. mono
pH	**1**															
TS	0.23	**1**														
VSS	0.22	**0.85**	**1**													
COD	0.12	**0.94**	**0.80**	**1**												
TKN	0.06	**0.79**	**0.66**	**0.79**	**1**											
NH_4^+	0.25	0.09	0.15	0.19	0.13	**1**										
VFA	0.15	-0.30	-0.29	-0.27	-0.25	-0.09	**1**									
PO_4^{3-}	0.30	0.03	0.07	-0.16	-0.24	-0.14	-0.10	**1**								
Mg^{2+}	0.28	0.04	0.05	-0.01	0.02	-0.20	0.09	**0.43**	**1**							
Ca^{2+}	0.39	0.20	0.24	0.22	0.15	**0.79**	-0.20	0.16	0.27	**1**						
K^+	-0.09	0.20	0.15	0.14	0.21	-0.01	-0.02	0.19	**0.46**	0.31	**1**					
Na^+	0.03	-0.05	-0.08	-0.13	0.01	-0.02	0.13	0.18	**0.53**	0.30	**0.89**	**1**				
Cu	-0.03	**0.90**	**0.78**	**0.94**	**0.77**	0.17	-0.30	-0.19	-0.19	0.10	0.03	-0.27	**1**			
Zn	0.00	**0.91**	**0.78**	**0.94**	**0.75**	0.20	-0.30	-0.17	-0.19	0.14	0.02	-0.28	**1.00**	**1**		
temp[a]	**0.79**	0.17	0.10	-0.04	0.00	0.04	0.05	**0.48**	0.29	0.31	0.28	0.38	-0.18	-0.16	**1**	
L. mono[b]	0.13	0.08	**0.46**	0.04	0.09	-0.07	-0.06	-0.14	-0.01	-0.11	-0.13	-0.09	0.07	0.04	-0.02	**1**

[a] Temperature; [b] *L. monocytogenes*; [c] Numbers in bold indicate a significant correlation ($\alpha = 0.05$)
